# Calcium signaling as a mediator of cell energy demand and a trigger to cell death

**DOI:** 10.1111/nyas.12885

**Published:** 2015-09-16

**Authors:** Gauri Bhosale, Jenny A. Sharpe, Stephanie Y. Sundier, Michael R. Duchen

**Affiliations:** ^1^ Department of Cell and Developmental Biology and Consortium for Mitochondrial ResearchUniversity College LondonLondonUnited Kingdom

**Keywords:** calcium signaling, mitochondrial calcium uptake, cell death, MICU1, mitochondrial permeability transition pore, ischemia/reperfusion injury

## Abstract

Calcium signaling is pivotal to a host of physiological pathways. A rise in calcium concentration almost invariably signals an increased cellular energy demand. Consistent with this, calcium signals mediate a number of pathways that together serve to balance energy supply and demand. In pathological states, calcium signals can precipitate mitochondrial injury and cell death, especially when coupled to energy depletion and oxidative or nitrosative stress. This review explores the mechanisms that couple cell signaling pathways to metabolic regulation or to cell death. The significance of these pathways is exemplified by pathological case studies, such as those showing loss of mitochondrial calcium uptake 1 in patients and ischemia/reperfusion injury.

## Introduction

Mitochondrial function is crucial for energy provision and health in higher eukaryotes, particularly in excitable cells, such as neurons, and in skeletal and cardiac muscle. Bioenergetic homeostasis, which ensures that energy supply has the capacity to meet energy demand, is key to normal cell and tissue health. Calcium signaling plays a central role in both communicating increased energy demand and in driving mechanisms that increase energy supply.

Calcium is a universal secondary messenger, playing a central role in a remarkably wide range of cellular processes, including muscular contraction, fertilization, synaptic transmission, cell migration and motility, and cell proliferation.^1^ Disordered cytosolic calcium [Ca^2+^]_(c)_ signaling plays a critical role in cell death. Mitochondria engage in an intricate and complex dialogue with calcium signals. The capacity of mitochondria to accumulate calcium was established many years ago through the work of the pioneers of mitochondrial biology, including Lehninger, Carafoli, and Attardi.^2^, ^3^, ^4^ More recent research has placed this work into the context of cell biology—the molecular identities of the major components of these pathways have been elucidated, and our understanding of the role of calcium signaling in health and disease has grown immensely in a short period of time. Since the pioneering work of Rizzuto *et al*.^5^ and of other researchers in the early 1990s,^6^, ^7^, ^8^, ^9^ it has become clear that most physiological calcium signals are associated with mitochondrial calcium uptake. This association is characterized as a true dialogue, in that calcium signals shape mitochondrial bioenergetic function, while mitochondrial calcium accumulation serves as a fixed spatial buffer with the capacity to shape the spatiotemporal patterning of calcium signals.

**Figure 1:**
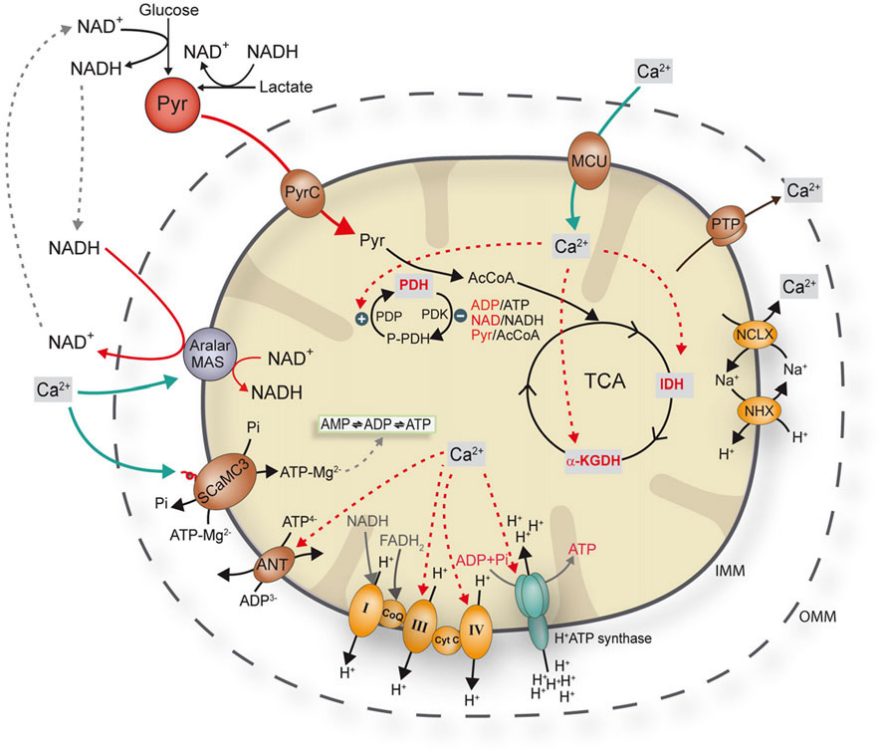
A schematic representation of the effect of calcium influx and efflux in mitochondria on respiration. Reproduced, with permission, from Llorente Folch *et al*.^83^

Calcium signals directly drive an increase in mitochondrial bioenergetic efficiency through two major pathways (Fig. 1), including one that is mediated by the calcium‐sensitive carrier SCaMC‐3, acting at the outer face of the inner mitochondrial membrane (IMM) and therefore responding directly to changes in [Ca^2+^]_(c)_. The other pathway, mediated by the mitochondrial calcium uniporter (MCU), requires a rise in matrix Ca^2+^ concentration ([Ca^2+^]_(m)_). The two pathways operate in concert to increase oxidative phosphorylation in response to cellular calcium signals. Thus, a rise in [Ca^2+^]_(m)_ concentration drives the allosteric activation of the three rate‐limiting enzymes of the tricarboxylic acid cycle: pyruvate, isocitrate, and α‐ketoglutarate dehydrogenase.^10^ The *V*_max_ of pyruvate dehydrogenase, isocitrate dehydrogenase, and α‐ketoglutarate dehydrogenase is enhanced upon Ca^2+^ binding, upregulating the production of reduced nicotinamide adenine dinucleotide (NADH) and resulting in a subsequent increase in mitochondrial membrane potential (Δψ_m_), followed by an increase in adenosine triphosphate (ATP) production. This was demonstrated directly in sensory neurons, where a calcium‐dependent increase in NADH fluorescence was observed upon stimulation^8^ and was also shown to directly increase ATP generation following calcium signals associated with fertilization in the mammalian oocyte.^11^ These mechanisms underlie a simple and elegant direct coupling of ATP demand to increased ATP supply mediated by calcium signaling.

A rise in [Ca^2+^]_(c)_ acts as a positive effector of Ca^2+^‐binding mitochondrial carriers (CaMCs), including aspartate/glutamate carriers (AGCs) and ATP‐Mg/P_i_ carriers (SCaMCs). Aralar and citrin are mammalian AGCs involved in the malate–aspartate NADH shuttle, which promotes the transfer of NADH from the cytosol to the mitochondria, thus providing reducing power for ATP generation via oxidative phosphorylation while also keeping the cytosolic NADH/NAD^+^ ratio low, favoring generation of pyruvate and promoting mitochondrial substrate supply. Cells overexpressing aralar and citrin showed a larger increase in mitochondrial ATP levels upon calcium stimulation compared to control cells, while mutants lacking Ca^2+^‐binding sites failed to show a significant energetic response to a calcium signal.^12^ The ATP‐Mg/Pi carriers are capable of modulating adenine nucleotide levels in mitochondria by exchanging cytosolic ATP‐Mg^2+^ for mitochondrial P_i_ in response to hormone‐induced calcium signaling.^13^, ^14^ Therefore, mitochondrial metabolism is integrated with calcium signaling by the actions of both [Ca^2+^]_(m)_ and [Ca^2+^]_(c)_. Interestingly, these also operate over different concentration ranges: the S_0.5_ for the activation of aralar by calcium is approximately 300 nM,^15^ while the intramitochondrial dehydrogenases are activated in the micromolar range. As mitochondrial calcium uptake is potential dependent and the potential will amplify the rise in calcium concentration, modest increases in cytosolic calcium are accompanied by significantly greater increases in matrix calcium concentration.

## Mechanisms for mitochondrial calcium uptake

**Figure 2:**
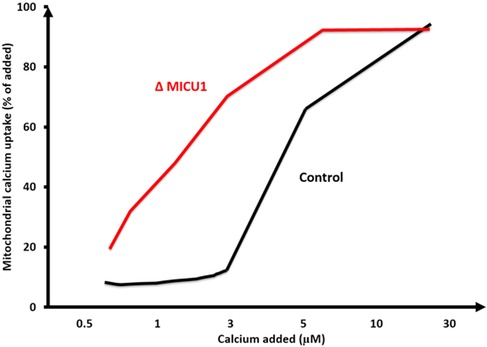
A schematic of the sigmoidal dependence of mitochondrial calcium uptake on added calcium. MICU1 interacts with the MCU through its N‐terminal polybasic domain and establishes a threshold of intracellular calcium concentration for mitochondrial calcium uptake.^31^, ^84^ The loss of MICU1 alleviates the threshold for uptake, meaning mitochondria are calcium loaded at rest and uptake occurs in a more linear fashion. Adapted, with permission, from Csordas *et al*.^32^

Mitochondrial calcium uptake depends on the electrochemical potential of Ca^2+^, dictated by the Δψ_m_ and by a low calcium concentration, which is maintained by a Na^+^/Ca^2+^ exchanger (NCX). While the outer mitochondrial membrane (OMM) is permeable, some studies have suggested that the voltage‐dependent anion channel (VDAC) in the OMM might act as a Ca^2+^‐activated Ca^2+^ channel.^16^, ^17^ The rise of [Ca^2+^]_(m)_ measured as a function of extramitochondrial Ca^2+^ exhibits a sigmoidal relationship, with a threshold at approximately 2–3 μM^18^ (Fig. 2). These characteristics reflect the properties of the MCU, which mediates the electrogenic movement of calcium through the selectively permeable IMM.

Although mitochondrial calcium uptake was first documented in the 1960s, the MCU was not identified until 2011.^19^, ^20^ The MCU consists of two highly conserved transmembrane domains that are predicted to oligomerize and form a tetrameric gated ion channel in the IMM.^21^ While its overexpression sensitizes cells to apoptotic stimuli, silencing of the MCU has no effect on mitochondrial respiration, membrane potential, or basic morphology,^19^, ^20^ which may seem surprising given its role in modulating energy demands. However, a recent study showed that MCU knockdown in pancreatic islet cells lowered the expression of components of the electron transport chain,^22^ suggesting that the role of the MCU is to mediate the transfer of peak [Ca^2+^]_(c)_ signals to the mitochondrial matrix with little effect on resting [Ca^2+^]_(m)_.

Interestingly, it is possible to create viable mice in which the MCU is globally knocked out (MCU‐KO) (although only when using an outbred strain^23^). Despite a complete lack of capacity for mitochondrial calcium accumulation, the MCU‐KO mice showed no change in basal metabolism, mitochondrial morphology, autophagy, or protection from cell death.^24^ The only reported physiological difference between MCU‐KO mice and their wild‐type littermates (besides the KOs being smaller than the wild‐type mice) is that the MCU‐KO mice showed reduced maximal skeletal muscle power output, suggesting that altering matrix calcium *in vivo* may be important in response to exercise.^24^ It is also worth noting that, in the MCU‐KO mice, mitochondrial calcium responses to stimulation were reduced but not completely abolished, suggesting the activity of other mechanisms mediating calcium uptake. In contrast, recent work with cardiac‐specific deletion of the MCU demonstrated that the MCU is required for cardiac ischemia/reperfusion injury via irreversible mitochondrial permeability transition pore (mPTP) opening. In addition, the MCU is involved in the regulation of bioenergetic supply in response to acute cardiac stress.^25^, ^26^ Therefore, recent results point to a role for the MCU in supporting bioenergetic demand of cells and tissues.

Alongside the channel‐forming MCU, a number of regulatory proteins have been identified that form part of a mitochondrial uniporter complex, and their exact stoichiometry, role, and interaction within the complex are still under scrutiny. The MCU paralog MCUb (previously known as CCDC109b) has been found to act as a dominant negative subunit of the MCU channel. The MCU/MCUb ratio appears to vary across different tissues, potentially providing a molecular mechanism to regulate the efficiency of mitochondrial calcium uptake.^21^ Mitochondrial calcium uptake regulator 1 (MCUR1; previously known as CCDC90A) was reported to be essential for MCU function, as its knockdown diminished mitochondrial calcium uptake and compromised cellular bioenergetics.^27^ However, recent work showed that MCUR1 does not directly regulate the MCU, is involved with the assembly of complex IV, and alters mitochondrial calcium uptake as a consequence of a decrease in mitochondrial membrane potential.^28^ Another proposed player in this regulatory network is SLC25A23, an Mg/ATP‐Pi carrier in the IMM, which, as reported by Hoffman *et al*.,^29^ interacts with the MCU and mitochondrial calcium uptake 1 (MICU1), and silencing of this carrier reduced MCU‐mediated mitochondrial calcium uptake.

MICU1 physically interacts with MCU and shares a similar tissue expression pattern.^19^ When initially characterized, MICU1 was described as indispensable for mitochondrial calcium uptake;^30^ however, subsequent studies have found this not to be the case, and it is now widely agreed that MICU1 acts as a gatekeeper of the MCU by setting the threshold concentration of intracellular calcium for mitochondrial calcium uptake.^31^, ^32^ By sensing calcium levels with its two EF hands, MICU1 keeps the MCU closed at low [Ca^2+^]_(c)_ (<3 μM) but allows cooperative activation of the channel as [Ca^2+^]_(c)_ exceeds the threshold (Fig. 2).

Although MCU‐KO mice may lack an obvious phenotype, the physiological importance of mitochondrial calcium handling has been highlighted by research on the first human disease to be associated with dysfunctional mitochondrial calcium uptake. Individuals with loss‐of‐function mutations in *MICU1* present with a brain and muscle disorder characterized by muscle weakness, learning difficulties, and progressive extrapyramidal motor disturbances.^33^ Typical features associated with mitochondrial disease were also reported in some patients, including ataxia, microcephaly, ophthalmoplegia, ptosis, optic atrophy, and axonal peripheral neuropathy. Surprisingly, no patients were reported to have diabetes mellitus, deafness, or cardiomyopathy. Available muscle biopsies showed myopathic features, including clusters of regenerating fibers and minicores.

**Figure 3:**
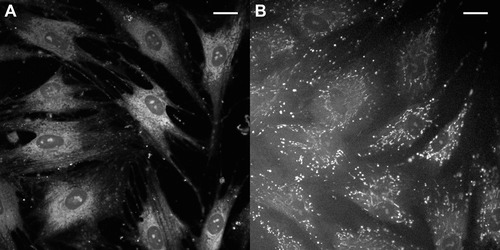
The loss of MICU1 results in increased basal mitochondrial calcium concentrations. Representative confocal image of Rhod2‐AM–labeled control (A) and MICU1‐KO (B) fibroblasts. Scale bar indicates 20 μm.

Fibroblasts from two individuals lacking MICU1 (hereafter referred to as MICU1‐KO) display a striking mitochondrial phenotype; their mitochondria are largely fragmented and calcium loaded, even at basal unstimulated cytosolic calcium concentrations (Fig. 3). The sigmoidal dependence of mitochondrial calcium uptake on calcium concentration is also lost in these cells, consistent with the previously characterized gatekeeping function of MICU1. Despite demonstrating chronic activation of the MCU, fibroblasts from MICU1‐KO show no change in mitochondrial membrane potential or oxygen consumption. The cells appear to have developed compensatory mechanisms to cope with such mitochondrial stress, which is perhaps reflected in the slow progression of clinical features in affected individuals,^33^ but it is clearly imperative to characterize function in the cell types primarily affected by the mutation: neurons and muscle. The neuromuscular phenotype of this mutation could be attributed to the effects of aberrant calcium signaling on calcium‐dependent processes, such as synaptic transmission and muscular contraction. These are also tissues that undergo major changes in energy demand on excitation and where reduced capacity to respond to changing demand may have the greatest effect on function.

The identification of neuromuscular disease associated with impaired mitochondrial calcium handling highlights an aspect of mitochondrial calcium signaling that tends to be ignored, which is that mitochondrial calcium accumulation carries an energetic cost. An increase in matrix Ca^2+^ inevitably leads to activation of efflux pathways, most notably the Na^+^/Ca^2+^ exchange. This increase will raise matrix Na^+^, which will in turn activate mitochondrial Na^+^/H^+^ exchange, bringing protons back into the matrix through a route that bypasses the ATP synthase, consuming proton motive force to balance calcium influx at the expense of ATP synthesis. In patients, it seems probable that the continuous transfer of Ca^2+^ into the matrix even at resting [Ca^2+^]_(c)_ is sufficient to drive a futile cycle where calcium is brought into the matrix and (indirectly) exchanged for protons, undermining the bioenergetic capacity of the cell. These observations illuminate the physiological role of MICU1, which serves as a signal‐to‐noise discriminator for mitochondrial calcium signaling and protects mitochondria from energetically wasteful basal calcium uptake.

## Mitochondrial permeability transition pore

Pathological and excessive mitochondrial calcium uptake is a major trigger for cell death and is mediated by the mPTP. Opening of the mPTP has been implicated in cell death associated with ischemia/reperfusion injury,^34^, ^35^ neurodegenerative disorders such as Alzheimer's disease and Parkinson's disease,^36^ several forms of muscular dystrophy and myopathy,^37^ and diabetes.^38^ Indeed, it seems to be a final common step in the pathway to necrotic cell death. Although permeability transition was first described in the 1970s in a series of seminal experiments carried out by Haworth and Hunter,^39^, ^40^, ^41^ it has only been in the last two decades that the role of the mPTP has been intensely studied in both necrotic and apoptotic cell death. Permeability transition reflects the formation of a nonspecific high‐conductance channel in the IMM, which allows unrestricted movement of solutes up to 1.5 kDa in size,^39^, ^42^, ^43^ resulting in the collapse of Δψ_m_, mitochondrial swelling due to the osmotic influx of water, and induction of ATP hydrolysis by the F_o_F_1_ ATPase. Mitochondrial swelling can cause rupture of the OMM, releasing proapoptotic factors, such as cytochrome *c*, apoptosis‐inducing factor, and procaspases from the intermembrane space. It has been suggested that a reversible low‐conductance state of the mPTP might play a physiological role in calcium signaling,^44^ while the high‐conductance state results in an irreversible mitochondrial catastrophe. Transient opening of the mPTP has been implicated in calcium‐induced calcium release from mitochondria, likely preventing mitochondrial calcium overload.^45^ In particular, the role of the mPTP in myocardial preconditioning–induced protection has been well documented.^46^, ^47^, ^48^

Pore opening is primarily triggered by increased [Ca^2+^]_(m)_, as well as by oxidative stress, increased phosphate levels, loss of Δψ_m_, and ATP depletion, which can lower the Ca^2+^ threshold required to trigger pore opening. Importantly, pore opening is limited by cyclosporine A (CsA), an immunosuppressant drug in widespread clinical use. Therefore, the mPTP represents an exciting pharmacological target with translational potential. A modulator of the mPTP was identified by studies that demonstrated that the archetypal mPTP inhibitor, CsA, worked through the inhibition of a matrix peptidyl‐prolyl *cis–trans* isomerase (PPIase), cyclophilin D (CypD).^49^, ^50^ Apart from cognitive dysfunction due to decreased neurotransmission,^51^ CypD gene knockout mice show normal development and almost no notable phenotype, raising questions about the physiological role of CypD. However, mitochondria isolated from CypD‐deficient mice reveal reduced sensitivity to pore opening in response to increased [Ca^2+^]_(m)_ and oxidative stress. Nonetheless, mPTP opening was not completely abolished and could be provoked by a higher [Ca^2+^]_(m)_.^35^

The molecular identity of the mPTP is still subject to debate. The VDAC and adenine nucleotide translocase (ANT) were among the first candidate components of the pore, as pharmacological modulators of ANT alter the probability of pore opening and the ANT can adopt channel‐like behavior in response to excess calcium.^52^ An mPTP complex comprising VDAC, ANT, and regulatory kinases was proposed on the basis of reconstitution experiments in liposomes.^53^ However, this model has not endured the scrutiny of genetic studies. Specifically, independent knockout experiments in mice suggested that neither ANT nor VDAC is essential for mPTP formation.^54^, ^55^ He and Lemasters^56^ proposed an alternative mechanism for pore formation, proposing that damage by oxidative stress caused misfolding of membrane proteins, which formed amphipathic clusters regulated by chaperones such as CypD. A role for the proposed regulator in the OMM, the translocator protein (TSPO), was again ruled out, as conditional knockout of the gene did not alter sensitivity to permeability transition in ischemia/reperfusion injury.^57^ Given the role of inorganic phosphate as an inducer of permeability transition, it is not surprising that the phosphate carrier (PiC), through its interactions with ANT and CypD, has been proposed as another candidate component of the mPTP.^58^ Again, cardiac‐specific overexpression or knockdown in mice failed to alter the mPTP response,^59^ and genetic ablation studies in mice suggest that the PiC is not essential for pore formation and may have a regulatory function instead.^60^

More recently, it has been postulated that the F_1_F_o_ ATP synthase may be the missing link in the composition of the mPTP complex. A study by Bernardi and colleagues^61^ suggested that dimers of the ATP synthase formed channels with similar properties as the mPTP and also showed that CypD binds to the oligomycin sensitivity–conferring protein (OSCP) subunit of the F_1_F_o_ ATP synthase. Two independent studies provided evidence to support a role of the c‐ring subunit of the F_1_F_o_ ATP synthase in the permeability transition. In one of these studies, knockdown of the c‐subunit resulted in HeLa cells being more resistant to mPTP activation.^62^ Consistent with these data, Alavian *et al*.^63^ reported that depletion of the c‐subunit attenuated cell death in neurons, whereas overexpression of a mutant c‐subunit with increased conductance resulted in the opposite effect. These authors proposed that the pore might be formed from a channel in the c‐ring exposed during the physical uncoupling of the F_1_ and F_o_ subunits. While these new developments in the search for the identity of the pore seem promising, knockdown of any component of the intricate network of mitochondrial bioenergetic pathways has complex downstream consequences that complicate interpretation of the data. Detailed studies of the various regulatory components of the pore complex and their interactions are needed to fully exploit the pharmacological potential of the mPTP for combating disease.

## Mitochondrial PTP opening in an ischemia/reperfusion injury model

Despite challenges in defining the identity of, and specific conditions for, mPTP assembly in isolated mitochondria and cell‐based models, the most clearly defined role for pore opening is in the context of ischemia/reperfusion injury. The primary organs susceptible to mPTP‐mediated ischemia/reperfusion injury are the brain,^64^ kidney,^65^ liver,^66^ and heart,^67^ where mitochondrial health is necessary for normal function.

Restriction of oxygen and nutrient delivery during ischemia leads to progressive changes in cell physiology that establish conditions that favor opening of the mPTP at reperfusion. Thus, a switch to anaerobic metabolism causes intracellular acidosis.^68^ The decrease in cellular pH activates the Na^+^/H^+^ exchanger (NHX), which removes protons but starts to raise intracellular Na^+^ ([Na^+^]_(i)_). The rise in [Na^+^]_(i)_ is exacerbated by slowed or inactive Na^+^/K^+^ ATPase pumps as ATP is depleted and also stimulates the NCX to reverse, increasing intracellular calcium concentration ([Ca^2+^]_(i)_).^69^, ^70^ Therefore, there is a progressive increase in [Ca^2+^]_(i)_ in cells within an infarct, which is coupled with the depletion of ATP and adenine nucleotides, and a progressive rise in inorganic phosphate. These changes will ultimately culminate in necrotic—and minor peripheral apoptotic—cell death, if ischemia continues uninterrupted.^71^, ^72^

Although the ischemic period of ischemia/reperfusion injury induces significant intracellular changes, the transitory phase of starvation primarily sets the scene for mitochondrial dysfunction and does not by itself necessarily drive irreversible injury. Although primed, mPTP opening is prevented due to the low intracellular pH during ischemia,^73^ while mitochondrial calcium overload required to trigger pore opening is limited, as the mitochondrial membrane potential is inevitably dissipated by prolonged ischemia. Severe injury occurs during the immediate aftermath of reperfusion, when oxygen is reintroduced to the deprived region and oxidative damage is initiated, a process sometimes described as the oxygen paradox.^74^, ^75^

At reperfusion, the reintroduction of oxygen restores respiration, immediately restoring the mitochondrial membrane potential and so driving rapid mitochondrial calcium uptake. The rise in [Ca^2+^]_(c)_ may be further stoked by the generation of free radical species (reactive oxygen species, or ROS), which stimulate calcium release from sarcoplasmic stores.^76^ The increases in mitochondrial calcium uptake combined with ROS generation, along with restoration of physiological pH, are sufficient to open the mPTP, depolarizing and disabling mitochondrial ATP production, and causing cell death.

Prevention of mPTP opening is an important potential therapeutic target for the protection and preservation of cells, especially in nonproliferating cardiomyocytes and neurons. As with many complex cellular processes, numerous methods have been used to broach the chain of events that eventually lead to mPTP opening following ischemia/reperfusion injury. Primarily, these procedures have spanned preinjury priming events, from remote^77^ and local preconditioning, to postinjury salvaging measures including postconditioning^78^ and the use of antioxidants to neutralize excessive ROS production. The mechanisms by which preconditioning and postconditioning reduce infarction sizes are still unknown, although efforts have been made in investigating cell signaling reperfusion injury salvage kinase pathways and receptors such as adenosine A1/A3, angiotensin II type I (AT1), bradykinin, and δ‐opioid receptors,^79^ as well as SDF‐1α/CXCR4^80^ and exosome signaling.^81^

**Figure 4:**
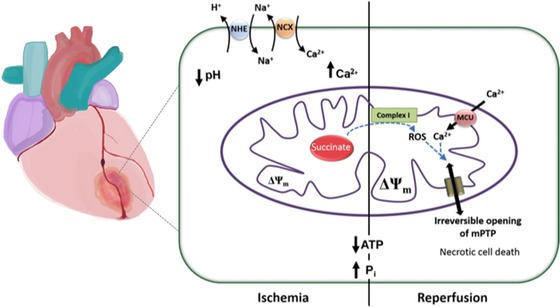
Pathological changes during ischemia and reperfusion in mitochondria, leading to necrotic cell death via mPTP opening in reperfusion.

Opening of the mPTP at reperfusion is a response to mitochondrial calcium overload in combination with high Pi, low ATP, and oxidative stress (Fig. 4). Recent work has identified the mechanisms involved in generating mitochondrial ROS at early reperfusion. Comparative metabolomic analysis of mouse tissues collected at ischemia and reperfusion revealed the universal ischemia‐specific accumulation of several metabolites, namely, xanthine, hypoxanthine, and succinate.^82^ Furthermore, pathway analysis revealed that succinate is generated through the metabolism of AMP through IMP to generate fumarate, which is converted to succinate at complex II of the respiratory chain.^65^ Measurements of ROS generation using confocal microscopy in isolated rat cardiomyocytes during ischemia and reperfusion (i.e., measurement of the rate of appearance of the fluorescent product of dihydroethidium oxidation) showed that the rate of ROS generation increased specifically at the onset of reperfusion and was modulated by the accumulation of succinate in mitochondria. Thus, incubation of cells with cell‐permeable dimethyl succinate increased the rate of ROS generation at reperfusion, while inhibition of complex II using dimethyl malonate—a competitive inhibitor of succinate dehydrogenase (SDH)—decreased the rate of ROS production upon reperfusion, as generation of succinate requires reversal of SDH to metabolize accumulated fumarate. At reperfusion, the reverse electron flow from SDH to complex I is driven by the high levels of succinate combined with limited capacity of the respiratory chain due to depletion of adenine nucleotides. That the reversed electron flow drives ROS generation at complex I was confirmed, as ROS generation was reduced by inhibition of complex I.

These experiments strongly suggest several potential novel therapeutic targets in the pathway to ROS generation that may help prevent mPTP opening and cell death following reperfusion. The field of mitochondrial biology faces the real prospect of combining strategies to salvage cardiac tissue—and likely tissues in other organ systems where the evidence is currently less developed—at risk for injury from ischemia.

## Conflicts of interest

The authors declare no conflicts of interest.
